# Online Multi-Parameter Identification for PMSM Parameter Monitoring Based on a ZOH Model and Dual-Sampling Strategy

**DOI:** 10.3390/s26031072

**Published:** 2026-02-06

**Authors:** Sidong He, Xuewei Xiang, Hui Li, Shuai Li, Peng Jiang

**Affiliations:** State Key Laboratory of Power Transmission Equipment Technology, School of Electrical Engineering, Chongqing University, Chongqing 400044, China; sidonghe@stu.cqu.edu.cn (S.H.); xueweixiang@cqu.edu.cn (X.X.); shuaili@stu.cqu.edu.cn (S.L.); jiangpeng@stu.cqu.edu.cn (P.J.)

**Keywords:** permanent magnet synchronous motor, zero-order hold, online identification, recursive least squares, nonlinear compensation

## Abstract

The accuracy of online parameter identification for permanent magnet synchronous motors (PMSMs) is constrained by discrete model errors, rank deficiency in the steady-state identification matrix, and voltage deviations resulting from inverter nonlinearities. This paper proposes a multi-parameter identification method acting as a high-precision virtual sensor, based on Zero-Order Hold (ZOH) discretization and an inverter nonlinear voltage compensation scheme utilizing a dual-sampling strategy. First, a discrete model of the PMSM, accounting for rotor position variations within the control period, is established using the ZOH discretization method. Compared with the forward Euler discretization method, this approach effectively minimizes discretization model errors, especially under high-speed operating conditions where rotor position variations are significant. Second, the rank deficiency problem of the steady-state identification matrix is overcome by combining d-axis small-signal injection with a dual-sampling strategy. Furthermore, the Forgetting Factor Recursive Least Squares (FFRLS) algorithm is introduced to achieve online multi-parameter identification. Finally, the influence mechanisms of the dead-time effect, power switch voltage drop, and turn-on delay on the output voltage are analyzed. Consequently, an inverter nonlinear voltage compensation strategy tailored for the dual-sampling mode is proposed. Experimental results demonstrate that the proposed method significantly enhances parameter identification accuracy across the entire speed range. Specifically, under high-speed conditions, the identification errors for resistance, inductance, and flux linkage are maintained within 5.47%, 4.05%, and 2.46%, respectively.

## 1. Introduction

With the rapid advancement of modern industry and technology, requirements for motor performance have become increasingly stringent. Permanent magnet synchronous motors (PMSMs) have found extensive application in aerospace, industrial automation, power generation, medical equipment, and transportation due to their superior performance characteristics, such as high efficiency, high power density, and excellent dynamic response [1,2]. Common PMSM control strategies, such as vector control, direct torque control (DTC), and model predictive control (MPC), rely heavily on fundamental motor parameters, including resistance, inductance, and permanent magnet flux linkage. However, in practical operation, motor parameters vary continuously with operating conditions such as temperature, magnetic saturation, and loading levels. This parameter mismatch degrades control performance and, in severe cases, may jeopardize closed-loop stability. In particular, apart from stator resistance and inductance, the permanent magnet flux linkage is significantly affected by the magnet’s temperature coefficient and thermal variations. Such variations can lead to flux linkage drift, thereby aggravating parameter mismatch and potentially undermining control performance and closed-loop stability [3,4]. Therefore, the precise online identification of motor parameters is not only essential for controlling system robustness but also serves as a crucial method for real-time condition monitoring and health diagnosis of the motor drive system [5,6].

Online parameter identification is typically based on the discrete mathematical model of the motor, estimating motor parameters in real time by acquiring electrical quantities such as voltage and current combined with algorithmic calculations. However, due to the limited switching frequency of insulated-gate bipolar transistors (IGBTs), the variation of rotor position within a single control period cannot be ignored when the PMSM operates at high speeds [7]. Under these conditions, the coupling between the d and q axes of the motor intensifies, and the error of the discrete motor model obtained based on traditional discretization methods increases significantly. This leads to reduced control accuracy of the current loop, slower dynamic response, and, in severe cases, system instability [8,9]. Therefore, to establish a precise PMSM model, Reference [10] utilized the volt-second equivalence principle combined with the forward Euler method to accurately model the process within a control period. Reference [11] constructed a new discrete prediction model by directly solving differential equations with the aid of an auxiliary coordinate system, which resulted in smaller control errors compared to the traditional Euler discrete model. Reference [12] pointed out that the digital pulse width modulation (PWM) Zero-Order Hold (ZOH) model possesses higher accuracy compared to the time-delay model and the Pade approximation model. Reference [13] established a discrete model considering the dynamic variation of the rotor position within the control period; however, there remains room for improvement regarding the handling of resistive voltage drop nonlinearity under complex operating conditions.

During the steady-state operation of PMSMs, the identification matrix becomes rank-deficient due to the linear dependence of input variables, causing the identified parameters to fail to converge [14]. To address rank deficiency, primary solutions include model reduction, rank augmentation via voltage vector combinations within a switching period, and signal injection. Model reduction strategies typically rely on reducing the number of target parameters or employing stepwise identification. The former relies on the assumption that the unidentified parameters are known and equal to their true values; however, this assumption is rarely satisfied in practice, and parameter coupling can readily lead to error propagation [15,16]. The latter relies on accurate initial values, and deviations in initial values will lead to significant identification errors [17]. Rank augmentation methods based on voltage vector combinations within a switching period face multiple challenges in practical applications: they impose high requirements on the sampling frequency, and due to the limited duration of effective vectors, reliable multiple sampling is difficult to achieve. Meanwhile, vector switching tends to induce current oscillation, further constraining the engineering practicality of this method [18]. Currently, the signal injection method is widely adopted, which includes rotor position signal injection and voltage/current signal injection. Although signal injection can achieve rank augmentation and improve parameter identification accuracy, excessive injection amplitude affects motor performance, generating torque ripple [19,20].

Direct measurement of inverter output voltage is often impractical; thus, the reference voltage is typically used. However, inverter nonlinearities, such as deadtime and voltage drops, introduce deviations that significantly degrade parameter identification accuracy [21,22]. Specifically, inverter nonlinear factors, such as the dead-time effect, IGBT, and the conduction voltage drop of power devices, cause deviations between the actual output voltage and the reference value, thereby severely constraining the accuracy of parameter identification. References [23,24] employed average voltage for parameter identification by judging the current polarity. However, the implementation of this compensation strategy relies on the real-time judgment of current polarity, and the zero-current clamping effect along with the nonlinearity at the current zero-crossing point severely restrict the accuracy of the compensation. Reference [25] proposed an improved dead-time compensation method based on a multiple cascaded extended state observer (MCESO). Although such advanced compensation algorithms can effectively estimate error voltages, they typically impose high requirements on the computational performance of the digital signal processor due to their complex algorithmic structures. Although Reference [26] achieved online look-up table compensation for inverter nonlinear error voltage, its compensation model depends on the accuracy of the offline model establishment and does not fully consider the nonlinear influence of parasitic capacitance on the switching process, leading to limited accuracy under actual operating conditions.

Addressing the aforementioned issues, this paper proposes a multi-parameter online identification scheme for PMSMs based on Zero-Order Hold (ZOH) discretization, which functions as a high-precision soft sensor for monitoring internal motor states. First, based on the digital PWM ZOH model, a discrete model of the PMSM considering the continuous variation of rotor position within the control period is established, laying a foundation for accurate parameter identification. Subsequently, to resolve the rank deficiency of the identification matrix during steady-state operation, a rank augmentation method combining d-axis current small-signal injection and a dual-sampling strategy is proposed. On this basis, the Forgetting Factor Recursive Least Squares (FFRLS) algorithm is introduced to achieve stable online identification of multiple parameters. Finally, to eliminate the voltage error introduced by inverter nonlinearities, the influence mechanisms of the dead-time effect, power device conduction voltage drop, and turn-on delay are analyzed, and a nonlinear voltage compensation strategy adapted for the dual-sampling scheme is proposed. Experimental results demonstrate that the proposed method exhibits significant advantages in parameter identification accuracy and stability under multiple operating conditions.

The main contributions of this paper are summarized as follows:

1. A high-precision discrete model considering rotor position variation within the control period is established based on the ZOH principle, which acts as the mathematical core of the virtual sensor to eliminate discretization errors at high speeds.
2. A robust identification scheme combining d-axis current injection and a dual-sampling strategy is proposed to solve the rank deficiency problem, ensuring the observability of all electromagnetic parameters.
3. An inverter nonlinearity compensation mechanism adapted for dual sampling is developed, which effectively suppresses the adverse effects of dead-time and voltage drops, improving the sensing accuracy of resistance and inductance parameters.

## 2. Online Multi-Parameter Identification for PMSMs Based on a Zero-Order Hold Discrete Model and Dual-Sampling Strategy

### 2.1. Zero-Order Hold Model of PMSM Considering Rotor Position Variations

To facilitate model derivation and online parameter identification, the Park transformation is employed to express the PMSM equations from the stationary three-phase abc reference frame in the synchronously rotating *d*-*q* frame. In this rotating frame, the steady-state stator voltages and currents become approximately DC quantities, and the inductance matrix becomes independent of the rotor position [27].

For surface-mounted permanent magnet synchronous motors (SPMSMs), where *L*_d_ = *L*_q_ = *L*, the mathematical equations in the synchronous rotating *d*-*q* reference frame can be expressed in matrix form as follows:

(1) $$\frac{d}{dt}i_{\mathrm{dq}}=Bi_{\mathrm{dq}}+Cu_{\mathrm{dq}}-De_{\mathrm{dq}}$$

where

(2) $$\begin{array}{c} i_{\mathrm{dq}}={\left[\begin{array}{cc} i_{d} & i_{q} \end{array}\right]}^{T}\;u_{dq}={\left[\begin{array}{cc} u_{d} & u_{q} \end{array}\right]}^{T}\;e_{\mathrm{dq}}={\left[\begin{array}{cc} 0 & \omega_{e} \end{array}\right]}^{T}\\ B=\left[\begin{array}{cc} -\frac{R}{L} & \omega_{e}\\ -\omega_{e} & -\frac{R}{L} \end{array}\right]\;C=\left[\begin{array}{cc} \frac{1}{L} & 0\\ 0 & \frac{1}{L} \end{array}\right]\;D=\left[\begin{array}{cc} \frac{\psi_{f}}{L} & 0\\ 0 & \frac{\psi_{f}}{L} \end{array}\right] \end{array}$$

**Figure 1:**
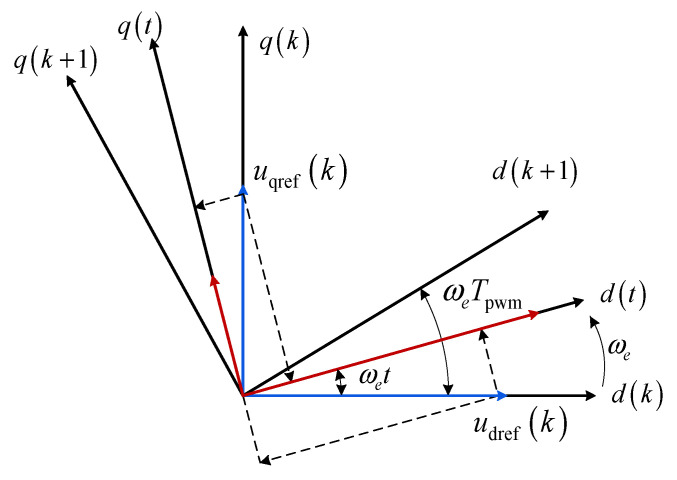
Actual voltage vector deviation caused by rotor displacement.

The time–space voltage vector trajectory of the PMSM is illustrated in Figure 1, where *T*_pwm_ denotes the pulse width modulation (PWM) period. The *d*-*q* coordinate system at the *k*-th instant is defined as *d*(*k*) and *q*(*k*). The voltage command ***u***_dq_(*k*) is applied starting from the instant *kT*_pwm_. Within one control period, the actual *d*-*q* coordinate axes rotate with the rotor position, causing the projection of the voltage vector ***u***_dq_(*k*) on the *d*-*q* axes to vary rather than remain constant. Based on Figure 1, the relationship between the actual voltage and the voltage command within one period can be expressed as

(3) $$u_{\mathrm{dq}}\left(k\right)=\left[\begin{array}{cc} \cos\omega_{e}t & \sin\omega_{e}t\\ -\sin\omega_{e}t & \cos\omega_{e}t \end{array}\right]u_{\mathrm{dqref}}\left(k\right)$$

By performing the Zero-Order Hold (ZOH) discretization and simplifying the expressions, the discrete model is obtained as

(4) $$i_{\mathrm{dq}}\left(k+1\right)=G_{\mathrm{ij}}i_{\mathrm{dq}}\left(k\right)+M_{\mathrm{ij}}u_{\mathrm{dqref}}\left(k\right)-F_{\mathrm{ij}}e_{\mathrm{dq}}\left(k\right)$$

The specific parameter expansions are given as follows:

(5) $$\begin{array}{l} G_{11}=G_{22}=e^{T\sigma }\cos\left(T\omega_{e}\right)\\ G_{12}=-G_{21}=e^{T\sigma }\sin\left(T\omega_{e}\right)\\ M_{11}=M_{22}=\frac{\left(\sigma \cos\left(2\omega_{e}T\right)e^{\sigma T}+2\omega_{e}\sin\left(2\omega_{e}T\right)e^{\sigma T}-\sigma \right)}{L\left(\sigma^{2}+4{\omega_{e}}^{2}\right)}\\ M_{12}=-M_{21}=\frac{\left(\sigma \sin\left(2\omega_{e}T\right)e^{\sigma T}-2\omega_{e}\cos\left(2\omega_{e}T\right)e^{\sigma T}+2\omega_{e}\right)}{L\left(\sigma^{2}+4{\omega_{e}}^{2}\right)}\\ F_{11}=F_{22}=\frac{\psi_{f}\left(\sigma \cos\left(\omega_{e}T\right)e^{\sigma T}+\omega_{e}\sin\left(\omega_{e}T\right)e^{\sigma T}-\sigma \right)}{L\left(\sigma^{2}+{\omega_{e}}^{2}\right)}\\ F_{12}=-F_{21}=\frac{\psi_{f}\left(\sigma \sin\left(\omega_{e}T\right)e^{\sigma T}-\omega_{e}\cos\left(\omega_{e}T\right)e^{\sigma T}+\omega_{e}\right)}{L\left(\sigma^{2}+{\omega_{e}}^{2}\right)} \end{array}$$

where *σ* = −*R*/*L*, and ***G***_ij_ denotes the element in the *i*-th row and *j*-th column of matrix ***G***_ij_. Similarly, ***M***_ij_ and ***F***_ij_ represent the corresponding elements of matrices ***M***_ij_ and ***F***_ij_, respectively.

To achieve parameter decoupling and reconstruction, and to isolate the resistance, inductance, and flux linkage parameters, a linear regression equation suitable for online identification is derived. After performing a first-order Taylor expansion of the matrix terms in Equation (4), the terms related to the resistance, inductance, and flux linkage can be separated from the voltage equation and treated as unknowns to be identified, while the remaining terms are incorporated into the voltage equation and regarded as known quantities. Furthermore, the known terms are constructed into a regressor vector and the unknown terms are grouped into a parameter vector, thereby enabling online identification of the resistance, inductance, and flux linkage parameters, as shown in Equation (6).

(6) $$\begin{array}{l} \left[\begin{array}{c} i_{d}\left(k+1\right)-\Delta_{d}\left(k\right)-\left(i_{d}\left(k\right)\cos T\omega_{e}\left(k\right)+i_{q}\left(k\right)\sin T\omega_{e}\left(k\right)\right)\\ i_{q}\left(k+1\right)-\Delta_{q}\left(k\right)-\left(i_{q}\left(k\right)\cos T\omega_{e}\left(k\right)-i_{d}\left(k\right)\sin T\omega_{e}\left(k\right)\right) \end{array}\right]=A_{h}\left(k\right)X=\\ \;\;\left[\begin{array}{c} T\left(i_{d}\left(k\right)\cos T\omega_{e}\left(k\right)+i_{q}\left(k\right)\sin T\omega_{e}\left(k\right)\right)\\ T\left(i_{q}\left(k\right)\cos T\omega_{e}\left(k\right)-i_{d}\left(k\right)\sin T\omega_{e}\left(k\right)\right) \end{array}\right.\left.\begin{array}{cc} Tu_{\mathrm{dref}}\left(k\right) & Te_{d}\left(k\right)\\ Tu_{\mathrm{qref}}\left(k\right) & Te_{q}\left(k\right) \end{array}\right]{\left[\begin{array}{ccc} \sigma & \frac{1}{L} & \frac{\psi_{f}}{L} \end{array}\right]}^{T} \end{array}$$

where Δ_d_(k) and Δ_q_(k) are intermediate variables defined as

(7) $$\begin{array}{c} \left[\begin{array}{c} \Delta_{d}\left(k\right)\\ \Delta_{q}\left(k\right) \end{array}\right]=\left[\begin{array}{cccc} u_{\mathrm{dref}}\left(k\right) & u_{\mathrm{qref}}\left(k\right) & e_{d}\left(k\right) & e_{q}\left(k\right)\\ u_{\mathrm{qref}}\left(k\right) & -u_{\mathrm{dref}}\left(k\right) & e_{q}\left(k\right) & -e_{d}\left(k\right) \end{array}\right]\\ {\left[\begin{array}{cccc} M_{11}-\frac{T}{L} & M_{12} & F_{11}-\frac{\psi_{f}T}{L} & F_{12} \end{array}\right]}^{T} \end{array}$$

In the calculation process, *M*_11_, *M*_12_, *F*_11_, and *F*_12_ are computed using Equation (5). Thus, a concise and linearly separable PMSM parameter identification model is obtained.

By utilizing the identification method described in the following section, the term 1/L is first obtained. Subsequently, this value is substituted into the expressions for σ and *Ψ*_f_ to calculate the resistance and flux linkage parameters.

### 2.2. PMSM Parameter Identification Based on Dual Sampling

During the steady-state operation of the motor, the number of observation equations is fewer than the number of parameters to be identified, resulting in a rank-deficient identification system. To address this issue, this paper constructs persistent excitation conditions by injecting a fixed-frequency small sinusoidal current signal into the d-axis, combined with a dual-sampling strategy, thereby resolving the rank deficiency problem in the identification process.

**Figure 2:**
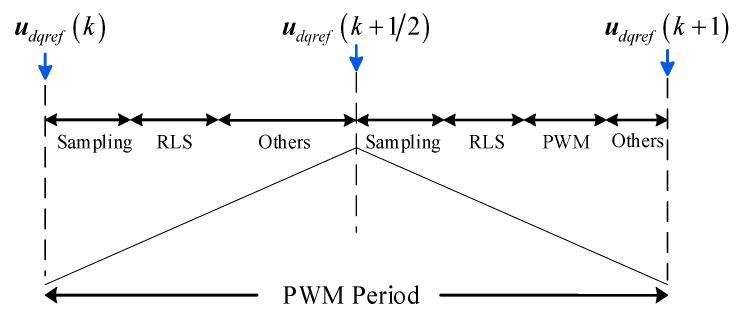
Dual-sampling timing diagram.

As illustrated in Figure 1, when the PMSM operates at medium to high speeds, the errors of *u*_d_ and *u*_q_ within one period become significant. Let the midpoint current and voltage of the k-th PWM period be denoted as ***i***_dq_ (k + 1/2) and ***u***_dq_ (k + 1/2), and the coefficient matrix formed by them be ***A***_h_ (k + 1/2). Combined with the sinusoidal signal injection, it is straightforward to prove that ***A***_h_ (k) and A_h_ (k + 1/2) are linearly independent, thus allowing a unique solution for the coefficient matrix ***X*** to be obtained, while simultaneously improving identification accuracy. The motor control timing diagram within one period is shown in Figure 2. Consequently, stacking the two equations within one PWM period can yield a regressor with improved rank property; together with the injected *d*-axis excitation, the resulting identification matrix can satisfy the full-column-rank condition required for unique parameter estimation.

The values of the midpoint current ***i***_dq_ (k + 1/2) and midpoint voltage ***u***_dq_ (k + 1/2) can be obtained from Equation (8):

(8) $$\begin{array}{l} i_{\mathrm{dq}}\left(k+\frac{1}{2}\right)=Ti_{\mathrm{abc}}\left(k+\frac{1}{2}\right)\\ u_{\mathrm{dqref}}\left(k+\frac{1}{2}\right)=Z\left(\frac{T_{\mathrm{pwm}}}{2}\right)u_{\mathrm{dqref}}\left(k\right) \end{array}$$

where matrix ***T*** is the *abc*-to-*dq* transformation matrix based on the angle *θ_e_* + *δ*, and *δ* = *ω_e_*(*k*) × *T_pwm_*/2. Substituting this into Equation (6) solves the rank deficiency problem.

In practical digital drives, the sampling and actuation chains introduce delays, which may cause an angle mismatch between the reference voltage and the actually applied voltage, as well as between the measured electrical quantities and their ideal sampling instants. In our implementation, such delays are mainly determined by the PWM time base, ADC conversion, and the fixed computation pipeline, and they can be regarded as nearly constant once the hardware and control flow are configured. Specifically, the delay at the start instant is approximately 1.5 *T*_pwm_, and the delay at the midpoint instant is approximately 0.75 *T*_pwm_. Therefore, the delay value can be accurately obtained by theoretical timing analysis and verified by simulation debugging, and then compensated in the angle update and the voltage generation stages. In this work, the proposed dual-sampling scheme is implemented with the above delay compensation, and the following analysis assumes that the control delay has been properly compensated.

Addressing the requirement for real-time identification of system parameters, the Forgetting Factor Recursive Least Squares (FFRLS) algorithm offers an online solution. Through recursive calculation, this algorithm continuously utilizes new data to correct estimation results, ultimately causing the parameter estimates to converge to the true values. This method features a low computational burden and fast convergence speed. The core calculation process of FFRLS can be expressed as

(9) $$\begin{array}{l} P_{k-1}=P_{k-1}/\lambda \\ P_{k}=P_{k-1}-\frac{\left(P_{k-1}a_{k}^{T}\right)\left(a_{k}P_{k-1}\right)}{1+a_{k}P_{k-1}a_{k}^{T}}\\ w_{k}=w_{k-1}+P_{k}a_{k}^{T}\left(y_{k}-a_{k}w_{k-1}\right) \end{array}$$

where ***P*** is the covariance matrix, *λ* is the forgetting factor, ***a***_k_ is the input vector for the *k*-th period, ***w*** is the estimated parameter vector for the *k*-th period, and ***y***_k_ is the output for the *k*-th period.

## 3. Nonlinear Voltage Error Analysis and Compensation Strategy

### 3.1. Nonlinear Modeling and Compensation Strategy Based on Dual Sampling

Influenced by inverter nonlinear characteristics such as the dead-time effect, power switch conduction voltage drop, and switching times, the inverter output voltage often exhibits nonlinear errors. If the command voltage is directly used as the input for the identification model, it will severely degrade the accuracy of online parameter identification. Therefore, establishing a precise inverter nonlinear error model and implementing compensation is crucial.

**Figure 3:**
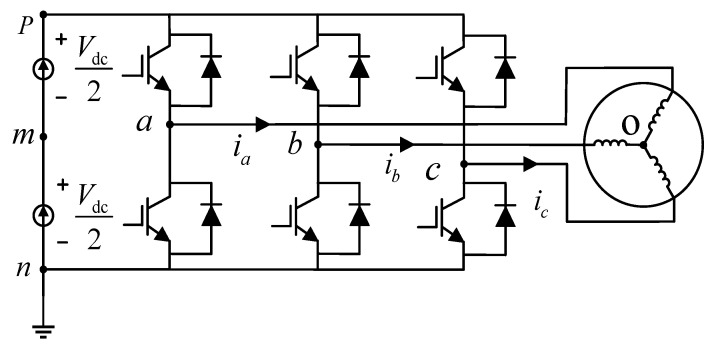
Three-phase voltage source inverter (VSI) topology.

The three-phase voltage source inverter (custom-built at Chongqing University, Chongqing, China) drive system of the PMSM (Guangzhou CNC Equipment Co., Ltd. (GSK), Guangzhou, China) is illustrated in Figure 3. Here, *V*_dc_ represents the DC-bus voltage; *p* and *n* denote the positive and negative DC-link terminals, respectively; *m* denotes the midpoint of the DC-link; *o* denotes the PMSM neutral point; and the current sign is defined as positive when flowing into the motor.

**Figure 4:**
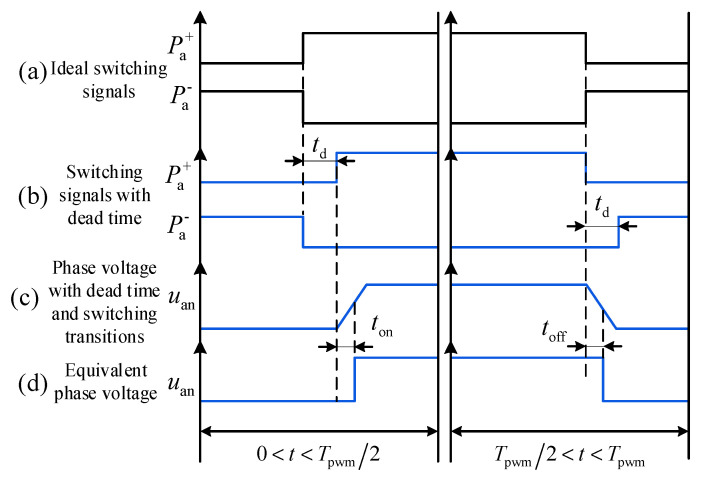
Dead-time effect based on dual sampling when *i*_a_ > 0.

Taking phase *a* as an example, the influence of the dead-time effect under the condition of stator current *i*_a_ > 0 is shown in Figure 4. In the figure, $P_{a}^{+}$ and $P_{a}^{-}$—represent the drive signals for the upper and lower arms of phase *a*, respectively; *u_an_* denotes the voltage between point *a* of the bridge arm and point *n* in Figure 3. The upper arm conducts at instant *t*_1_, and the lower arm conducts at instant *t*_2_. *t*_on_ and *t*_off_ are the turn-on delay and turn-off delay of the IGBT, respectively, while *t*_d_ represents the dead time. Figure 4a displays the switching signal waveforms of phase *a* under ideal conditions. When *i*_a_ > 0, to avoid simultaneous conduction of the upper and lower IGBTs, as shown in Figure 4b, the upper switch is not turned on immediately when the lower switch is turned off. During the dead time, the current flows as indicated by the blue arrows in Figure 4, at which point the freewheeling diode of the lower arm begins to conduct. Subsequently, the upper switch conducts at instant *t*_1_ + *t*_d_. Similarly, after the upper arm IGBT turns off at instant *t*_2_, the lower arm turns on after a delay of *t*_d_.

Figure 4c illustrates the actual output voltage waveform of phase *a* considering the IGBT turn-on/off delays and voltage rise/fall edges. By equivalencing the voltage rise and fall edges in Figure 4c to turn-on/off delays, the equivalent output voltage waveform shown in Figure 4d is obtained. At this time, the actual conduction time of the upper arm is *t*_2_ + *t*_off_ − (*t*_1_ + *t*_d_ + *t*_on_), whereas the ideal conduction time is *t*_2_ − *t*_1_. Based on the above analysis, defining the conduction time error as *T*_err_, it can be obtained by the following equation:

(10) $$T_{\mathrm{err}}=\left\{\begin{array}{c} -t_{d}-t_{\mathrm{on}}\;,\;\mathrm{for}\;t<T_{\mathrm{pwm}}/2\\ t_{\mathrm{off}}+0,\;\mathrm{for}\;t>T_{\mathrm{pwm}}/2 \end{array}\right.$$

**Figure 5:**
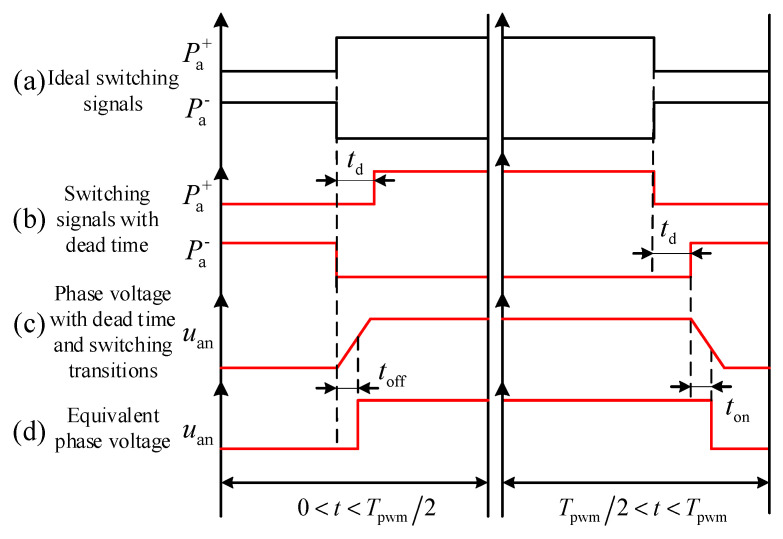
Dead-time effect based on dual sampling when *i*_a_ < 0.

When *i*_a_ < 0, the influence of the dead-time effect is illustrated in Figure 5.

Similarly, by analyzing the scenario where *i*_a_ < 0, the conduction time error, denoted as *T*_err_, can be derived as

(11) $$T_{\mathrm{err}}=\left\{\begin{array}{c} 0-t_{\mathrm{off}}\;,\;\mathrm{for}\;t<T_{\mathrm{pwm}}/2\\ t_{d}+t_{\mathrm{on}}\;,\;\mathrm{for}\;t>T_{\mathrm{pwm}}/2 \end{array}\right.$$

According to the analysis in Reference [28], let the command phase voltage for phase *a* be $u_{\mathrm{ao}}^{*}$ and the actual value be *u*_ao_. Considering the nonlinear effects shown in Figure 4a, the voltage error *u*_aoerr_ within one period is calculated as

(12) $$\begin{array}{c} \begin{array}{c} u_{\mathrm{aoerr}}=u_{\mathrm{ao}}-u_{\mathrm{ao}}^{*}=\frac{V_{\mathrm{dc}}}{3T_{\mathrm{pwm}}}\left(2\Gamma_{a}-\Gamma_{b}-\Gamma_{c}\right)\\ \;\;\;+\frac{1}{6}\left(-V_{ce}-V_{d}\right)\left[2sign\left(i_{a}\right)-sign\left(i_{b}\right)-sign\left(i_{c}\right)\right] \end{array}\\ \Gamma_{j}=\left\{\begin{array}{cc} t_{\mathrm{off}} & \;,\;\mathrm{for}\;i_{j}>0\;\mathrm{and}\;t>\frac{T_{\mathrm{pwm}}}{2}\\ -t_{d}-t_{\mathrm{on}} & ,\;\mathrm{for}\;i_{j}>0\;\mathrm{and}\;t<\frac{T_{\mathrm{pwm}}}{2}\\ -t_{\mathrm{off}} & ,\;\mathrm{for}\;i_{j}<0\;\mathrm{and}\;t<\frac{T_{\mathrm{pwm}}}{2}\\ t_{d}+t_{\mathrm{on}} & ,\;\mathrm{for}\;i_{j}<0\;\mathrm{and}\;t>\frac{T_{\mathrm{pwm}}}{2} \end{array}\right.\;j=\left(a,b,c\right) \end{array}$$

where *V*_ce_ represents the voltage drop of the IGBT, and *V*_d_ represents the forward voltage drop of the freewheeling diode. The auxiliary variable *Γ_j_* (*j*∈{a, b, c}) is a time quantity representing the equivalent conduction-time error of phase *j* caused by the dead time *t*_d_, the device turn-on delay *t*_on_ and turn-off delay *t*_off_. Here, *t*∈(0, *T*_pwm_) denotes the time instant within one PWM period, and the piecewise definition of *F_j_* depends on the current polarity *i*_j_ and the sampling instant *t* within one PWM period. Sign(·) is the sign function, i.e., sign(*i*) = 1, if *i* > 0, and sign(*i*) = −1 if *i* < 0. Based on the aforementioned analysis, the voltage errors for phase b and phase c within a single period can be derived in a similar manner. The resulting three-phase error voltages are then transformed into the *d*-*q* reference frame to compensate for the voltage references *u*_dref_ and *u*_qref_ in Equation (6).

Functioning as a software-based calibration for the virtual sensor, this strategy corrects voltage deviations to ensure high-fidelity inputs, effectively preventing error propagation in parameter estimation.

### 3.2. Processing Mechanism for Current Zero Crossing in Discrete Systems

**Figure 6:**
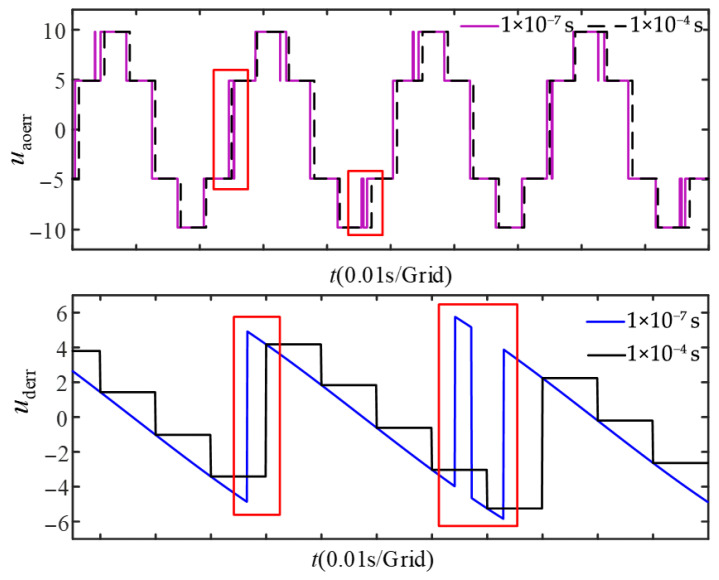
Compensation voltage under two simulation step sizes.

The calculations for the compensation voltage described above are performed under continuous conditions and do not account for the discretization characteristics of the digital control system. Based on Equation (12), the simulation results of the compensation voltage under different sampling periods are shown in Figure 6. The system with a sampling period of 1 × 10^−7^ s is approximated as a continuous system, while the system with a sampling period of 1 × 10^−4^ s is regarded as the actual discrete control system. Taking the phase a and d-axis compensation voltages as examples, in the continuous system, the compensation voltage exhibits abrupt changes near the zero-current point. Therefore, as highlighted in the boxed regions of Figure 6, the finite sampling period of the digital control system makes it difficult for the discrete implementation to capture the fast current variations within one control period, and sampling errors near the zero-current point further increase the deviation of the calculated compensation voltage. In particular, during the zero-crossing commutation interval, the conduction path switches between the IGBT and the freewheeling diode, causing the compensation terms to change abruptly with current polarity and leaving residual compensation errors. To mitigate this effect, a current-amplitude gate *i*_lim_ is introduced in the proposed scheme to reduce the influence of zero-crossing samples on online identification.

**Figure 7:**
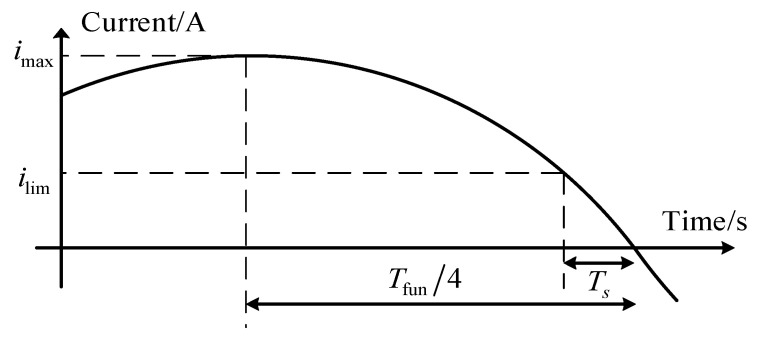
Current sampling timing diagram.

To mitigate the quantization errors inherent in discrete control systems near zero-crossing points, this paper proposes a robust data selection strategy. This strategy filters out low-confidence data segments where current polarity is ambiguous, ensuring that the FFRLS algorithm operates solely on high-quality excitation signals, thereby enhancing the overall robustness of the identification. Let the amplitude of the three-phase current be *i*_max_, which equals the *L*_2_ norm of the *d*-*q* axis currents. The period of the three-phase current is *T*_fun_, which can be obtained from the electrical angular velocity *ω*_e_. Considering the limit case, the phase current waveform and the sampling period timing diagram can be represented as shown in Figure 7. From the figure, *i*_lim_ can be derived as

(13) $$i_{\lim}=i_{\max}\sin\left(\omega_{e}T_{s}\right)$$

When the current magnitude is small around the zero crossing, the measured polarity is easily affected by noise or quantization, which may corrupt the regressor and the FFRLS update. Therefore, the identification update is performed only when the absolute value of the phase current *i_x_* (where *x* = *a*, *b*, *c*) satisfies |*i_x_*| ≥ *i*_lim_; otherwise, the update is skipped to improve robustness.

**Figure 8:**
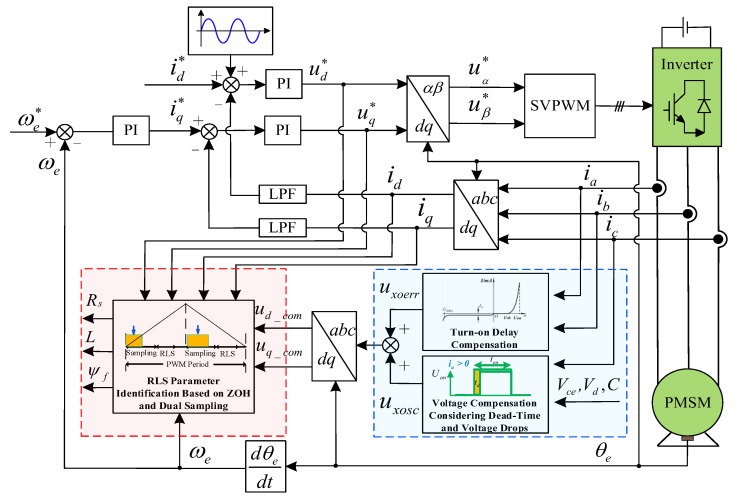
System control block diagram.

The overall system control block diagram is shown in Figure 8. The sampled three-phase currents *i*_abc_ are input into the nonlinear compensation module to calculate the compensation voltage, accounting for time delays, dead-time effects, and voltage drops. The sampled three-phase currents and the compensation voltage undergo coordinate transformation to obtain the *d*-*q* axis currents and *d*-*q* axis compensation voltages. These are then input into the dual-sampling-based RLS parameter identification block, which utilizes the Zero-Order Hold (ZOH) discretization model. Ultimately, the online identification of the three parameters—resistance, inductance, and permanent magnet flux linkage—is achieved.

## 4. Experimental Results and Analysis

**Figure 9:**
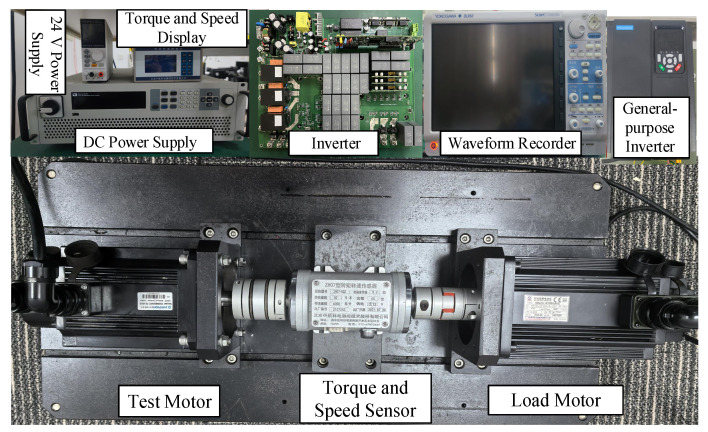
Experimental platform.

An experimental platform, illustrated in Figure 9, was established to validate the proposed virtual sensor strategy for online parameter identification. The test rig features a 2.5 kW PMSM with 4 pole pairs as the subject motor. The digital control system was implemented using a TMS320F28377D digital signal processor (DSP, Texas Instruments (TI), Dallas, TX, USA) and was equipped with a 17-bit absolute encoder (built-in, Guangzhou CNC Equipment Co., Ltd. (GSK), Guangzhou, China) to acquire high-precision rotor position and speed information. To simulate various operating conditions, the load system utilizes a second coaxially coupled PMSM, where torque loading is regulated through a general-purpose inverter.

The parameters of the PMSM used in the experiment are listed in Table 1. To obtain the true parameter values for algorithm evaluation, the stator resistance and inductance were measured multiple times using a high-precision LCR meter (UT622E, UNI-T, Dongguan, Guangdong, China), and the average values were reported. Specifically, the line-to-line inductance Lab(θ) was measured between terminals a and b, with terminal c left open while the rotor was slowly rotated. The measured results were averaged over rotor positions, and the stator phase inductance was calculated as L ≈ Lab/2. Since the subject motor is an PMSM where the magnetic circuit is isotropic Ld ≈ Lq, this measured value is adopted as the nominal d-q axis inductance listed in Table 1. The permanent magnet flux linkage parameter was calculated through the back-EMF amplitude and electrical angular velocity measured in a dragging no-load experiment.

**Figure 10:**
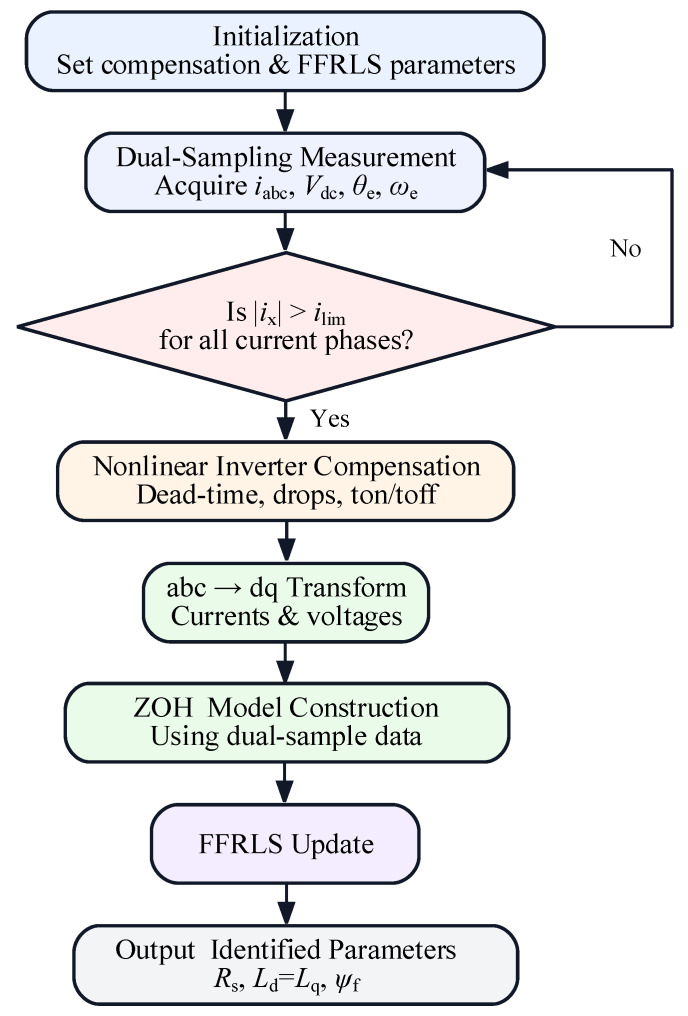
Flowchart of the online parameter identification algorithm.

As shown in Figure 10, the flowchart of the proposed online identification algorithm is presented. After initialization, in each control period, the system performs dual sampling to acquire the three-phase currents, DC-bus voltage, rotor electrical position, and electrical angular speed. Then, a decision is made on whether to perform the online parameter identification. When the absolute values of all phase currents are greater than the threshold *i*_min_ the algorithm enter s the nonlinear inverter compensation stage to correct the voltage errors caused by dead time and device nonidealities. The compensated voltages are transformed into the *d*-*q* reference frame, and a ZOH-based online identification model is constructed using the dual-sample data. Finally, the parameters are identified online via the FFRLS recursion and the identified results are output. If the gating condition is not satisfied, the update is skipped in the current period, and dual sampling and the threshold check are repeated in the next control period.

To evaluate the computational burden, the proposed routine was implemented on a TMS320F28377D with a 200 MHz system clock, and the execution time was measured using the on-chip TMS320F28377D timer. The measured total execution time of the identification routine is approximately 53 μs. With a PWM frequency of 4 kHz, the corresponding control period is 250 μs, indicating that the proposed identification consumes about 21% of the available computation time per period. Therefore, sufficient computational margin remains for other control tasks, such as current and speed regulation, demonstrating the feasibility of the proposed strategy for real-time implementation.

### 4.1. Comparative Experiment of Identification Algorithms and Result Analysis

To evaluate the identification accuracy of the proposed method over a wide speed range, comparative experiments were conducted at two representative operating points. A medium–low-speed case of 500 r/min was selected, where the signal amplitude is relatively small and the identification performance is more sensitive to discretization and measurement noise; a typical high-speed case of 2000 r/min was chosen, where the back EMF becomes dominant and the rotor position varies rapidly, making discretization-related errors more evident. At both speeds, the proposed approach was compared with a conventional ZOH-based method and a baseline method without dual sampling.

First, under the medium-high speed condition of 2000 r/min and 4 N·m, three methods were employed for parameter identification to perform a comparative analysis: (1) the proposed method (ZOH + dual-sampling compensation); (2) the ZOH method without compensation; and (3) the forward Euler method based on dual-sampling compensation. During the identification process, the rank deficiency problem was resolved by injecting a sinusoidal current signal with an amplitude of 0.5 A and a frequency of 10 Hz into the d-axis combined with dual sampling. The injection amplitude of 0.5 A was selected based on the experimental study reported in Section 4.2. The injection frequency was set to 10 Hz because an overly low frequency leads to only slight variations in the coefficients of the identification matrix, which may not provide sufficient excitation and can result in rank deficiency. In contrast, an overly high frequency increases current ripple and torque disturbance and reduces the effective number of current samples within one control period, which may introduce waveform distortion. Therefore, 10 Hz was adopted as a compromise.

**Figure 11:**
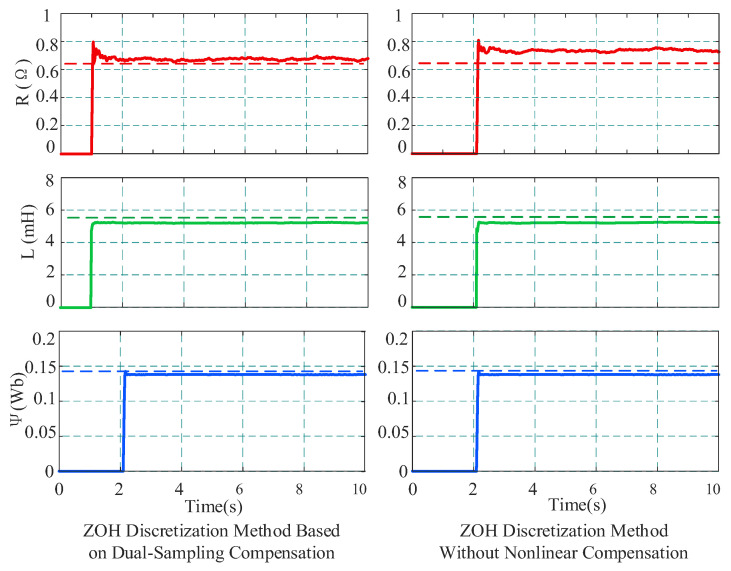
Comparison of identification results with and without compensation at 2000 r/min.

Figure 11 displays the parameter identification dynamic response and steady-state results under the load condition of 2000 r/min and 4 N·m. In Figure 11, dashed lines represent the offline calibrated true values, while solid lines represent the online identification results. Comparing the identification results of the two schemes reveals that when using the ZOH discretization identification without accounting for compensation, there are large deviations in the identification results. Specifically, the identified stator resistance value is 0.732 Ω, significantly higher than the true value; the inductance and flux linkage are 5.213 mH and 0.1378 Wb, respectively. When the proposed method is used for identification, the identification accuracy is significantly improved. The resistance converges to 0.675 Ω, the inductance to 5.278 mH, and the flux linkage to 0.1385 Wb. The experimental data indicate that under high-speed conditions, nonlinearities easily cause voltage distortion, thereby affecting identification accuracy.

**Figure 12:**
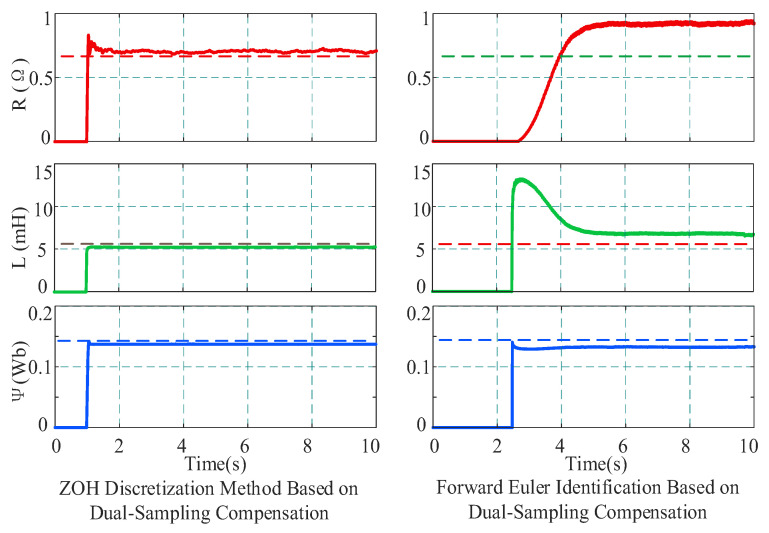
Comparison of identification results between ZOH and Euler discretization at 2000 r/min.

To verify the influence of the discretization model itself on algorithm performance, under the same condition of 2000 r/min and 4 N·m, this paper compared the proposed method with the forward Euler discretization identification method based on dual-sampling compensation. The comparison results are shown in Figure 12. Experimental results demonstrate that although both methods introduce the nonlinear compensation mechanism, the forward Euler method is limited by its discretization accuracy, resulting in significant deviations in identification results: the estimated resistance is 0.912 Ω, and the inductance and flux linkage are 6.498 mH and 0.1261 Wb, respectively, severely deviating from the true values. In contrast, the ZOH discretization strategy adopted in this paper can more accurately describe the discrete characteristics of the system, ensuring that parameters converge near the true values.

Table 2 summarizes the relative error data for the three schemes under the condition of 2000 r/min and 4 N·m. The relative errors of resistance are generally large for all methods; specifically, the error of the proposed method is 5.47%, while that of the forward Euler method reaches 42.50%. This is mainly because the baseline stator resistance of the tested motor is only 0.64 Ω, making the algorithm extremely sensitive to measurement noise and system disturbances, where a small absolute deviation causes a large relative percentage error. Conversely, the identification accuracy of the flux linkage parameter is generally high, with errors controlled within 3%. This is attributed to the back EMF dominating the voltage equation in the medium-high speed operation region; as the speed increases, the signal-to-noise ratio for flux linkage observation significantly enhances.

**Figure 13:**
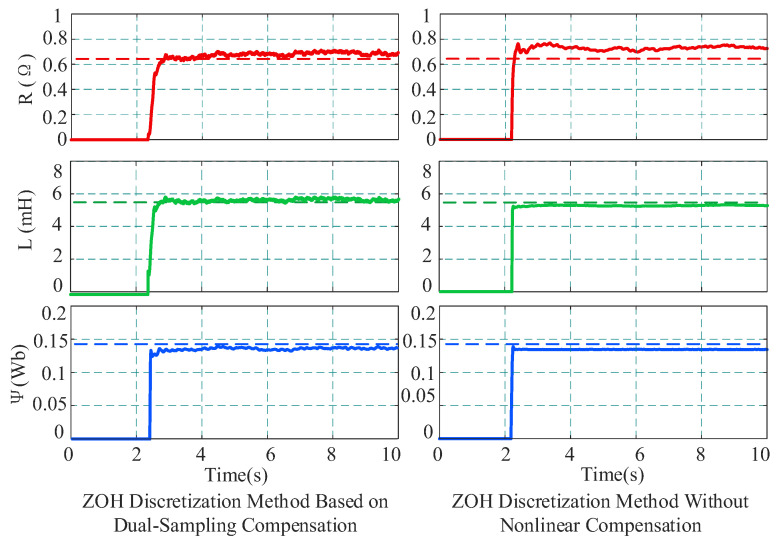
Comparison of identification results with and without compensation at 500 r/min.

To verify the applicability and identification accuracy of the proposed method in the medium–low-speed range, experiments were conducted at 500 r/min with a 4 N·m load under low-speed conditions, and the identification performance of different strategies was compared. The identification results are shown in Figure 13. The data indicate that when the proposed method is used, the parameter identification results are highly stable, with resistance converging to 0.673 Ω, and inductance and flux linkage converging to 5.561 mH and 0.1387 Wb, respectively, all extremely close to the offline calibrated values. In contrast, the ZOH method without nonlinear compensation yields a resistance of 0.713 Ω, an inductance of 5.363 mH, and a flux linkage of 0.1381 Wb. Although it converges, a certain degree of systematic bias remains. Comparing these results with the experimental data at 2000 r/min in Figure 11, it is observed that the identification accuracy of both methods improves slightly at 500 r/min. This is primarily because, under low-speed conditions, the ratio of the sampling frequency to the fundamental frequency increases significantly, thereby reducing approximation errors during the discretization process.

**Figure 14:**
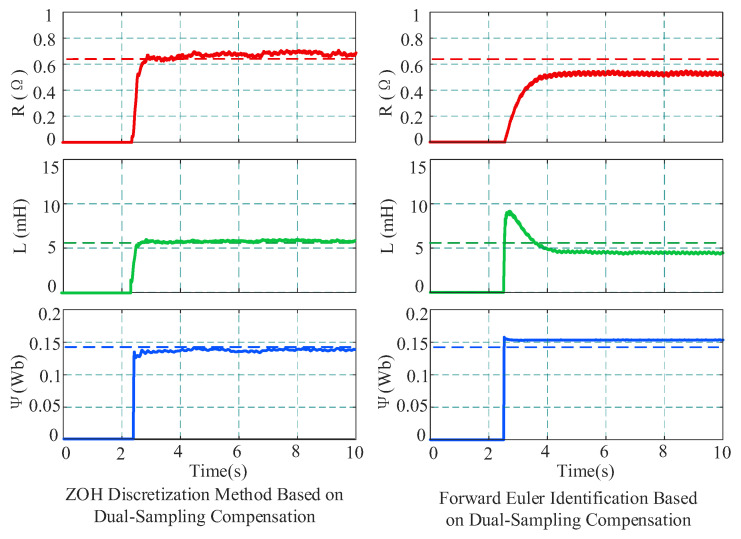
Comparison of identification results between ZOH and Euler discretization at 500 r/min.

At 500 r/min under 4 N·m load conditions, the comparison results between the proposed method and the identification method based on forward Euler discretization with dual-sampling compensation are shown in Figure 14. The identification results of the proposed method are consistent with those described above. However, the resistance identified by the forward Euler method with dual-sampling compensation is 0.528 Ω, the inductance is 4.375 mH, and the flux linkage is 0.1541 Wb. In contrast, the proposed method utilizes a high-precision ZOH discrete model, effectively reducing discretization errors and achieving precise identification of motor parameters.

The identification errors of the above three methods at 500 r/min and 4 N·m are summarized in Table 3. The error distribution reveals that, when comparing the two ZOH schemes, the resistance identification error decreases significantly from 11.41% to 5.16% after adopting the dual-sampling compensation strategy. This directly proves the necessity of dual-sampling compensation for improving identification accuracy in the low-speed region. Furthermore, a lateral comparison of different discretization methods demonstrates that the ZOH model used in this paper is comprehensively superior to the forward Euler method in terms of accuracy. In particular, the inductance error is optimized from as high as −20.45% to 1.11%, fully verifying the significant advantage of the proposed discretization model in enhancing modeling precision.

Synthesizing the experimental results from both the 2000 r/min and 500 r/min conditions, the proposed method exhibits superior identification performance at these representative operating points. By introducing accurate discrete modeling based on ZOH and a voltage compensation strategy based on dual sampling, this method not only ensures the correctness of the theoretical model but also effectively mitigates nonlinear disturbances from the hardware.

### 4.2. Analysis of the Influence of Different Operating Conditions on Identification Accuracy 

To comprehensively evaluate the adaptability and robustness of the proposed parameter identification method under complex operating conditions, this paper further conducted multiple sets of variable conditions. The effects of different motor speeds, load torques, and d-axis injected current amplitudes on the identification accuracy of the proposed method were comparatively analyzed.

**Figure 15:**
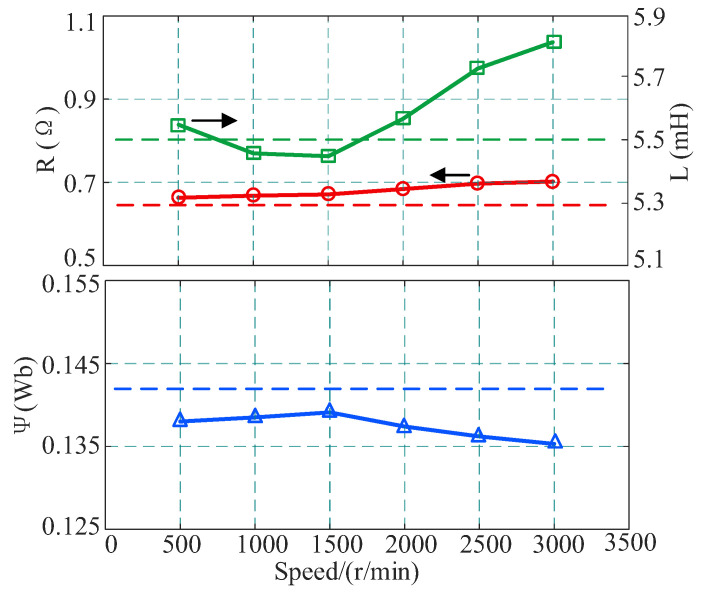
Parameter identification results at different speeds.

First, under a fixed load of 3 N·m and d-axis current injection of 0.5 A, the identification performance was tested during the process of increasing motor speed from 500 r/min to 3000 r/min. The results are summarized in Table 4 and Figure 15.

Experimental data show that as the speed increases, the identification errors of various parameters exhibit different trends. Both resistance and inductance identification errors show an upward trend with increasing speed. At the high-speed point of 3000 r/min, the resistance error reaches a maximum of 9.68%, and the inductance error is 5.74%. The main reason for this phenomenon is that at high speeds, the back-EMF term dominates the terminal voltage, causing the weights of the resistive voltage drop *Ri_dq_* and the inductive induced voltage *Ldi*/*dt* in the total voltage equation to decrease significantly. Consequently, the observability of these two parameters decreases, leading to a reduction in the identification accuracy of the algorithm. Unlike the former, the identification error of the flux linkage parameter remains below 5% across the entire speed range, with a minimum of only −2.04%. This benefits from the significant increase in back-EMF amplitude at high speeds, which greatly improves the signal-to-noise ratio of the flux linkage information, thereby verifying that the proposed method possesses strong robustness for flux linkage identification over a wide speed regulation range.

**Figure 16:**
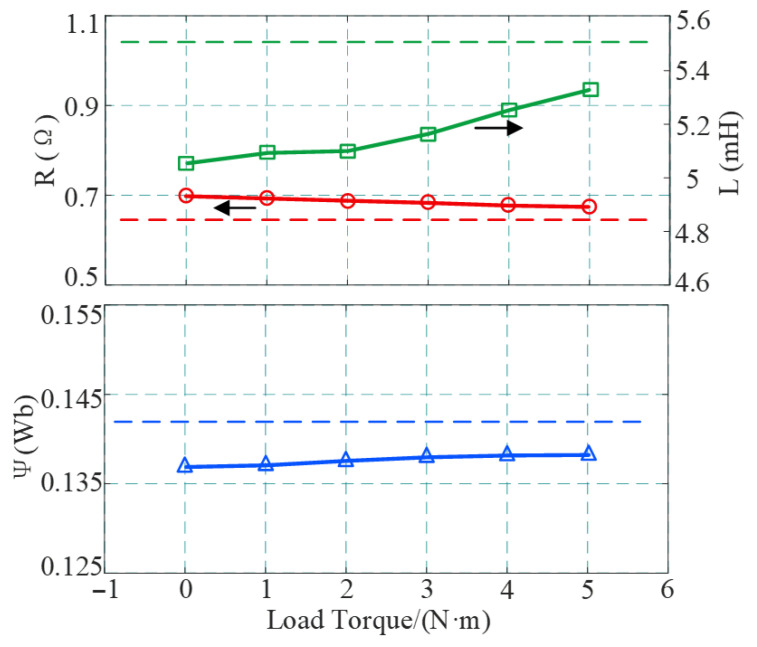
Identification results under different load torques.

To verify the influence of different load torques on identification accuracy, the identification results were tested under the condition of a constant motor speed of 1500 r/min, with the load torque varying from no-load (0 N·m) to full load (5 N·m). The data are summarized in Table 5 and Figure 16.

Through analysis, it is found that when the load varies within the range of 0–5 N·m, the overall fluctuation of parameter identification errors is small, indicating that the proposed method has good adaptability to different load conditions. However, increasing the load still has a positive impact on identification accuracy: the resistance error decreases from 8.59% at no-load to 4.84% at full load, and the inductance error also decreases from −8.10% to −3.12%. This is because the increase in load torque directly leads to an increase in the amplitude of the motor stator current. According to the motor voltage equation, the increase in current significantly strengthens the resistive voltage drop and the inductive induced voltage, thereby improving the accuracy of parameter identification.

To further validate the robustness of the proposed identification method under varying operating conditions, a load torque step experiment is conducted while keeping the speed reference unchanged. At 1500 r/min, when the load torque steps from 2 N·m to 5 N·m, the post transient estimates converge to *R* = 0.669 Ω, *L* = 5.338 mH, and *ψ* = 0.1380 Wb. Relative to the nominal values, the corresponding errors are approximately 4.53%, 2.95%, and 2.82%, indicating that the identification accuracy remains at a low error level. Moreover, the steady-state estimates under this step condition are consistent with those obtained in the steady-load tests. This is because, although the load step causes an abrupt operating point change and thus alters the regressor data sequence, the identification regressor matrix preserves the same rank property as in the injection stage and remains full rank due to the within-period injected excitation, thereby ensuring the identifiability and convergence of the parameter estimates. Consequently, the torque step only induces a brief adjustment in the parameter estimates, after which they stably converge to the same steady level as that in the steady-load tests.

**Figure 17:**
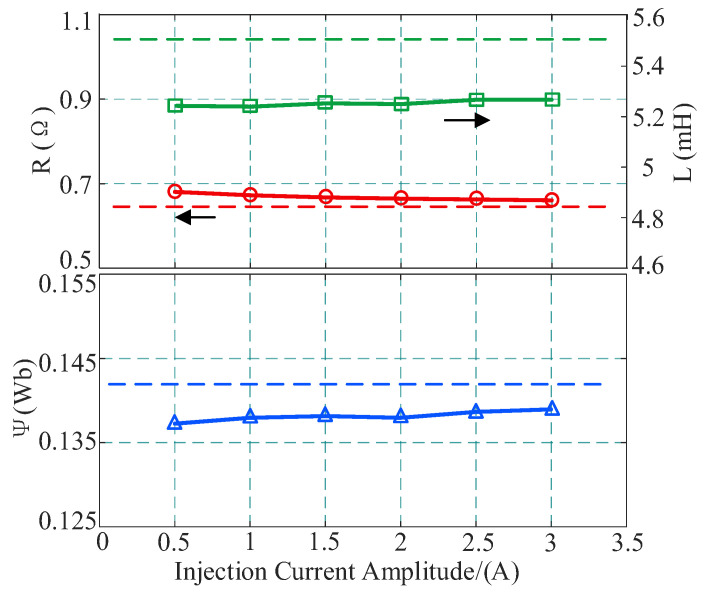
Identification results under different injection current amplitudes.

Finally, the influence of the amplitude of the *d*-axis injection signal on identification performance is analyzed. Under the condition of 1500 r/min, with a fixed injection frequency of 10 Hz, the amplitude of the injected current is gradually increased from 0.5 A to 3 A. The identification results and error distribution are shown in Table 6 and Figure 17.

Through comparative analysis, it is found that by applying an injection signal of only 0.5 A, the system can obtain sufficient persistent excitation to ensure the stable convergence of inductance and flux linkage parameters, with errors controlled within −4.78% and −3.38%, respectively. This strongly proves the effectiveness of the proposed strategy: a weak injection signal combined with the dual-sampling strategy is sufficient to solve the steady-state rank deficiency problem without introducing large-amplitude system disturbances. As the injection amplitude increases, the improvement in the identification accuracy of inductance and flux linkage is limited and exhibits a saturation characteristic. In contrast, the resistance identification error is more sensitive to this change, significantly decreasing from 6.40% to 3.28%. Although a larger current can slightly improve resistance accuracy, the associated risks of additional copper loss and torque ripple cannot be ignored. Therefore, this paper ultimately selects 0.5 A as the optimal injection amplitude, thereby achieving the best trade-off between identification performance and smooth motor operation.

It is noted that temperature variation mainly affects the stator resistance *R*_s_ and the PM flux linkage *ψ*_f_. Since each identification test in this paper is of relatively short duration, the temperature change during a single test is limited. Because the proposed scheme estimates *R*_s_ and *ψ*_f_ online, temperature-induced drift would appear as slow changes in the identified parameters; a systematic evaluation under wider temperature variations will be considered in future work.

Synthesizing the above comparative experiments regarding different speeds, load torques, and injection current amplitudes, the parameter identification method proposed in this paper demonstrates excellent identification accuracy and strong robustness under various operating conditions. Experimental results indicate that this method not only maintains high parameter identification accuracy within a wide speed regulation range and the full load region, effectively overcoming model mismatch and inverter nonlinear disturbances, but also requires only a weak injection signal to satisfy the full-rank excitation condition. This minimizes the disturbance to the system while ensuring identification accuracy, thereby achieving the optimal balance between identification precision and operational stability. The aforementioned experimental results strongly verify that this strategy is capable of adapting to complex and changing practical application scenarios, providing reliable parameter support for high-performance permanent magnet synchronous motor control systems.

## 5. Conclusions

This paper proposes a multi-parameter online identification method for permanent magnet synchronous motors (PMSMs) based on a Zero-Order Hold (ZOH) discrete model, functioning as a high-fidelity virtual sensor. By integrating precise discrete modeling, a dual-sampling strategy, and nonlinear voltage compensation, the system systematically improves its sensing performance. The following conclusions are drawn:

1. A high-precision discrete model accounting for rotor position variations is established based on the ZOH method, serving as the mathematical core of the virtual sensor. Compared with traditional approaches, this model effectively minimizes discretization errors, particularly in medium-to-high speed regions.
2. A robust identification strategy combining d-axis signal injection and dual sampling is implemented. This solution effectively resolves the rank deficiency problem of the identification matrix, ensuring the observability of electromagnetic parameters for stable real-time monitoring.
3. A nonlinear voltage compensation scheme is proposed as a software-based calibration mechanism. By correcting inverter distortions, it significantly enhances sensing accuracy and ensures robust parameter identification across the entire speed range.

In addition, several directions are worth further investigation. Future work will extend the proposed identification algorithm to IPMSMs and saturation conditions, where the inductances vary with current and operating point. Moreover, since the present nonlinear compensation may still suffer from unmodeled dynamics and parameter drift, improving its accuracy and adaptability will be pursued. In addition, the thermal dependence of the PM flux linkage will be systematically evaluated under wider temperature variations, and temperature-aware modeling and compensation will be explored by referring to Refs. [3,4]. Finally, the identification strategy will be integrated with higher-performance control schemes to enable online self-tuning of the control-loop parameters.

## Figures and Tables

**Table 1:** Parameters of the PMSM used in the experiment.

Parameter	Value	Parameter	Value
Rated Voltage/V	380	Pole Pairs	4
Rated Current/A	7.2	Stator Resistance/Ω	0.64
Rated Power/kW	2.5	*d*-*q* axis Inductance/mH	5.50
Rated Speed (r/min)	3000	Flux Linkage/Wb	0.142
Rated Torque/N·m	5	PWM Frequency/kHz	4

**Table 2:** Identification errors of cifferent methods at 2000 r/min and 4 N·m.

Parameters	ZOH (Proposed)	ZOH (No Comp)	Euler (Comp)
Resistance Error (%)	5.47	14.38	42.50
Inductance Error (%)	−4.05	−5.22	18.15
Flux Linkage Error (%)	−2.46	−2.96	−11.20

**Table 3:** Identification errors of different methods at 500 r/min and 4 N·m.

Parameters	ZOH (Proposed)	ZOH (No Comp)	Euler (Comp)
Resistance Error (%)	5.16	11.41	−17.50
Inductance Error (%)	1.11	−2.49	−20.45
Flux Linkage Error (%)	−2.32	−2.75	+8.52

**Table 4:** Identification results at different speeds.

Speed (r/min)	Resistance (Ω)		Inductance (mH)		Flux Linkage (Wb)	
	Value	Error (%)	Value	Error (%)	Value	Error (%)
500	0.663	3.59	5.556	1.01	0.1380	−2.81
1000	0.668	4.37	5.466	−0.61	0.1385	−2.46
1500	0.671	4.84	5.457	−0.78	0.1391	−2.04
2000	0.684	6.85	5.575	1.36	0.1374	−3.23
2500	0.697	8.90	5.733	4.23	0.1362	4.08
3000	0.702	9.68	5.816	5.74	0.1353	4.71

**Table 5:** Identification errors under different load torques.

Load Torque (N·m)	Resistance (Ω)		Inductance (mH)		Flux Linkage (Wb)	
	Value	Error (%)	Value	Error (%)	Value	Error (%)
0	0.695	8.59	5.054	−8.10	0.1368	−3.66
1	0.69	7.81	5.093	−7.40	0.137	−3.52
2	0.685	7.03	5.1	−7.27	0.1375	−3.16
3	0.68	6.25	5.164	−6.10	0.1379	−2.88
4	0.674	5.31	5.252	−4.50	0.1381	−2.74
5	0.671	4.84	5.328	−3.12	0.13816	−2.70

**Table 6:** Identification errors with different injection current amplitudes.

Injection Current Amplitude (A)	Resistance (Ω)		Inductance (mH)		Flux Linkage (Wb)	
	Value	Error (%)	Value	Error (%)	Value	Error (%)
0.5	0.681	6.40	5.237	−4.78	0.1372	−3.38
1	0.673	5.15	5.234	−4.83	0.1379	−2.88
1.5	0.668	4.37	5.247	−4.60	0.1381	−2.74
2	0.665	3.90	5.244	−4.65	0.1379	−2.88
2.5	0.663	3.59	5.261	−4.34	0.1386	−2.39
3	0.661	3.28	5.261	−4.34	0.1389	−2.18

## Data Availability

The data presented in this study are available on request from the corresponding author.
